# Validated frailty measures using electronic primary care records: a review of diagnostic test accuracy

**DOI:** 10.1093/ageing/afad173

**Published:** 2023-11-17

**Authors:** Carmen Brack, Mary Kynn, Peter Murchie, Stephen Makin

**Affiliations:** Centre for Rural Health, Institute of Applied Health Sciences, University of Aberdeen, Aberdeen, AB25 2ZD, United Kingdom; Institute of Applied Health Sciences, University of Aberdeen, Aberdeen, AB25 2ZD, United Kingdom; Academic Primary Care Group, Institute of Applied Health Sciences, University of Aberdeen, Aberdeen, AB25 2ZD, United Kingdom; Centre for Rural Health, Institute of Applied Health Sciences, University of Aberdeen, Aberdeen, AB25 2ZD, United Kingdom

**Keywords:** frailty, primary care, risk stratification, screening, systematic review, older people

## Abstract

**Introduction:**

Identification of people who have or are at risk of frailty enables targeted interventions, and the use of tools that screen for frailty using electronic records (which we term as validated electronic frailty measures (VEFMs)) within primary care is incentivised by NHS England. We carried out a systematic review to establish the sensitivity and specificity of available primary care VEFMs when compared to a reference standard in-person assessment.

**Methods:**

Medline, Pubmed, CENTRAL, CINHAL and Embase searches identified studies comparing a primary care VEFM with in-person assessment. Studies were quality assessed using Quality Assessment of Diagnostic Accuracy Studies revised tool. Sensitivity and specificity values were extracted or were calculated and pooled using StatsDirect.

**Results:**

There were 2,245 titles screened, with 10 studies included. These described three different index tests: electronic frailty index (eFI), claims-based frailty index (cFI) and polypharmacy. Frailty Phenotype was the reference standard in each study. One study of 60 patients examined the eFI, reporting a sensitivity of 0.84 (95% CI = 0.55, 0.98) and a specificity of 0.78 (0.64, 0.89). Two studies of 7,679 patients examined cFI, with a pooled sensitivity of 0.48 (95% CI = 0.23, 0.74) and a specificity of 0.80 (0.53, 0.98). Seven studies of 34,328 patients examined a polypharmacy as a screening tool (defined as more than or equal to five medications) with a pooled sensitivity of 0.61 (95% CI = 0.50, 0.72) and a specificity of 0.66 (0.58, 0.73).

**Conclusions:**

eFI is the best-performing VEFM; however, based on our analysis of an average UK GP practice, it would return a high number of false-positive results. In conclusion, existing electronic frailty tools may not be appropriate for primary care-based population screening.

## Key Points

- Proactive frailty identification by measures using electronic primary care records has been shown to be beneficial.
- This systematic review identified three such measures (electronic frailty index (eFI), claims-based frailty index and polypharmacy).
- However, the eFI would mis-identify several individuals compared to the frailty phenotype standard.
- Overall, there is scope to improve upon the diagnostic test accuracy of validated electronic frailty measures in primary care.

## Introduction

Early identification of frailty, defined as the state of being more vulnerable to physiological insult, may give the opportunity to prevent and reduce adverse health outcomes, including death, falls, disability and hospitalisation [1–5]. Therefore, proactively identifying those who are currently, or at risk of, becoming frail may lead to better clinical outcomes.

The English General Practitioners’ contract [6] has mandated the identification and appropriate management of those aged ≥65 with frailty since 2017. The most rigorously validated assessment tools are those which require in-person assessment [2]. However, it would not be practical to assess the whole over 65 population routinely in such a way. Practice electronic records are a valuable source of information of patients and offer an attractive solution as they may be utilised to automatically generate a list of patients who are most at risk of being or becoming frail. Such a list could then be used to offer earlier interventions, typically multicomponent in nature and individually tailored, but usually utilising some combination of exercise or nutrition enhancements [7].

We aimed to identify currently available primary care records-based validated electronic frailty measures (VEFM) which could be (or already are) utilised in the clinical setting and to assess their diagnostic accuracy in comparison to in-person assessment, following the Preferred Reporting Items for Systematic Reviews and Meta-Analyses (PRISMA) reporting guidelines [8]. To our knowledge, this is the first systematic review of diagnostic test accuracy focused on VEFMs. Previous reviews utilising diagnostic test accuracy methodology have focused on either simple measurements [9] or self-reported instruments [10]. We aim to add to the literature by broadening this to measures which use electronic health records as they are becoming more common.

## Methods

### Search strategy and selection criteria

#### Types of studies

We included primary care or community-based studies of adults, of any age, from any country and published in peer review journals in any language. We excluded studies which focused on those with a particular medical condition (e.g. frailty in those with cancer) to reduce the heterogeneity between studies and to allow for potential subgroup meta-analysis.

To be included in the review, studies needed to compare an in-person assessment of frailty (reference standard) with a VEFM (the index test). In-person assessment included any multifactorial assessment, such as the Frailty Phenotype (FP) or Frailty Index, for example, or a one-dimensional assessment, such as grip strength. The index test must be data-based using information that would be available to a primary care physician from electronic primary care records without requiring further virtual or face-to-face assessment of an individual. When data were collected for a population study, information must feasibly be available within primary care. The test must have a threshold to dichotomise patients into ‘frail’ and ‘not frail’. Where studies reported on multiple frailty assessments, we only included those which were relevant to our inclusion criteria (i.e. would be feasible to for a primary care physician to utilise using primary care records).

#### Selection of studies

We searched databases up to and including 4 May 2022. MEDLINE, Embase and the Cochrane Central Register of Controlled Trials were all searched in Ovid using the search terms given in Supplementary Material 1. The CINAHL search was a copy of the same strategy used in Ovid. In Pubmed, filters were applied to filter out any review papers. No language or date restrictions were applied (Supplementary Material 1).

Author C.B. then performed a forward citation search (identifying articles which cited these studies) on Google Scholar using the ‘cited by’ feature. The studies which referenced those we identified as relevant were then title, abstract and full-text screened in the same way as the electronic search. The database search results were also used to perform a search of the index frailty assessments found by searching, for example, ‘electronic frailty index’ (eFI) in Pubmed. These results were systematically title, abstract and full-text screened by C.B. and S.M. We also identified several previous systematic reviews, the references of which we searched [11–15].

### Data extraction and management

We piloted a data extraction form (Supplementary Material 2) on three studies (C.B.). We adapted this following discussion between both reviewers (C.B. and S.M.), considering the best presentation and potential analysis which would be possible. Then, the form was transferred into an Excel Spreadsheet to allow for ease of analysis.

### Assessment of methodological quality

Two reviewers (C.B. and S.M.) used the Quality Assessment of Diagnostic Accuracy Studies revised tool (QUADAS-2) [16] to assess the quality of included studies. This assesses the study quality on four domains: patient selection, index test, reference standard and flow and timing. A range of risk of biases were considered: how physical assessments handled patients who could not perform the test, whether the study was carried out on a cohort of patients generalised to UK primary care or only a subset of patients.

**Figure 1:**
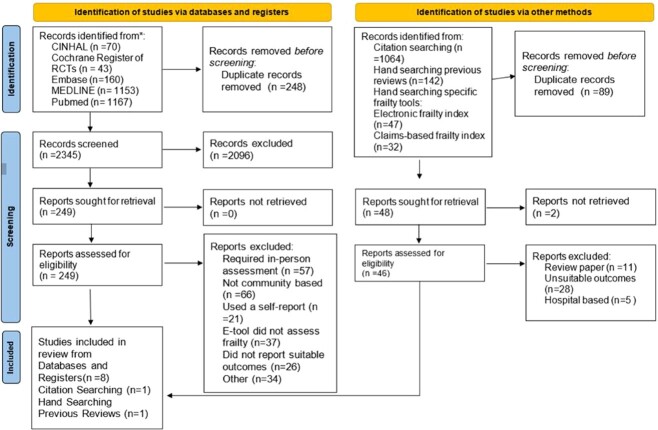
PRISMA [6] flow diagram; 2,245 studies were identified from databases and 1,064 were identified from other methods after the removal of duplicates. In total, 195 studies (149 from databases and 46 from other sources) were full-text screened for eligibility with reasons for exclusion detailed. 10 studies met the inclusion criteria for this review.

### Statistical analysis and data synthesis

We used the reported data to construct two-by-two tables in Review Manager 5.4.1 and to calculate the sensitivity and specificity; this is presented alongside the 95% confidence intervals. To allow for continuity of results, studies for which this was not possible (e.g. reported correlation analysis), pooling was done in subgroups, based on index test, using Stats Direct. The data were pooled using proportions to give an estimated overall sensitivity and specificity expected by the index test to allow for some comparison between them. A random effects model was used to due to the heterogeneity between studies, particularly considering age and frailty severity range of participants included and expected.

## Results

### Results of the search

A summary of the search results is shown in the PRISMA [8] flow diagram (Figure 1). The database search produced 2,345 citations, and 249 of these were deemed to be potentially eligible following title/abstract. Following further screening by the second reviewer (S.M.), 8 were included for the analysis; 32 of the 249 were used for citation searching, which produced 1,064 results; 1 paper was included via this route. Searching the included papers of previous reviews produced 60 results, with 1 paper being included.

Papers were excluded due to not being community-based (*n* = 66), having a self-report reference standard (*n* = 21), an index test which was based on in-person assessment (*n* = 57), not reporting suitable outcomes (*n* = 26) or for other reasons (*n* = 34). Overall, 10 papers are included in this review.

### Summary of results

Ten studies were included with a total of 42,067 participants (Table 1). The reference standard in all papers was the FP. In all papers, frailty was defined as the presence of three or more out of the five phenotype criteria. Where pre-frailty was reported, we categorised these as not frail individuals. The methods used to collect information of the five criteria varied; however, all conformed to the five standard Fried criteria of unintentional weight loss, weakness, exhaustion, slow walking speed and low physical activity [2]. The prevalence of frailty (by FP) ranged from 9.9 to 44.7% with a mean of 22.4% and was calculated as a total across all studies as 16.6%.

**Table 1:** Study characteristics

Study	Setting	Country	Study design	Index test	Reference standard	Sample size	Age (mean/SD)	Gender (% female)
Ambagtsheer 2019 [17]	GP clinic	Australia	Cross-sectional	eFI	FP	60	80.2 (4.8)	60%
Ambagtsheer 2020 [20]	3 GP clinics	Australia	Cross-sectional	Polypharmacy	FP	228	79.0 (6.0) (median/IQR)	55%
Festa 2020 [18]	Community (Health and Retirement Study)	USA	Cross-sectional	cFI	FP	3,097	75.7 (7.2)	58%
Herr 2015 [21]	Community (SIPAF Study)	France	Cross-sectional	Polypharmacy	FP	2,350	83.3 (7.5)	59%
Hoogendijk 2013 [22]	Single primary care practice (Identification of Frail Elderly Study in the Netherlands)	Netherlands	Cross-sectional	Polypharmacy	FP	102	78.6 (7.1)	57%
Jung 2020 [23]	Community (Korean Frailty and Aging Cohort Study)	Korea	Cross-sectional	Polypharmacy	FP	2,907	75.8 (3.9)	58%
Midão 2021 [24]	Community (SHARE Waves 6 and 7)	Europe	Cohort	Polypharmacy	FP	24,693	74.5 (6.9)	55%
Reallon 2020 [25]	Single Day-care unit	France	Cross-sectional	Polypharmacy	FP	403	80.5 (6.4)	57%
Saum 2017 [26]	Community (ESTHER Study)	Germany	Cohort	Polypharmacy	FP	3,058	69.6 (6.3)	52%
Segal 2017 [19]	Community (Cardiovascular health Study)	USA	Cohort	cFI	FP	4,582	75.2 (5.5)	59%

Three index tests were identified: the eFI [17], claims-based frailty index (cFI) [18, 19] and polypharmacy [20–26]. The most studied test was polypharmacy with seven papers; two papers were on the cFI, and one was on both EASY-Care TOS and eFI. Given that all studies used FP as their reference standard, we stratified analysis by index test.

## Findings

Table 2 summarises the reported sensitivity and specificity values that were either directly extracted from one study, in the case of the eFI [17], or were pooled using random effects proportion analysis for cFI [18, 19] and polypharmacy [20–26]. We stratified by the index test and these are described below.

**Table 2:** Summary of index test accuracy

**Index test**	**Cut-off**	**Reference standard**	**Sensitivity (95% CI)**	**Specificity (95% CI)**
eFI [17]	0.21	FP	0.84 (0.55,0.98)	0.78 (0.64,0.89)
Claims-based (pooled) [18, 19]	0.2	FP	0.48 (0.23,0.74)	0.80 (0.53,0.98)
Polypharmacy (pooled) [20–26]	≥5 medications	FP	0.61 (0.50,0.72)	0.66 (0.58,0.73)

### Electronic frailty index

There was a study (*n* = 60) that uses this tool similar to the Frailty Index, but the index items are taken from the primary care data alone rather than from the interview/assessment [17]. When compared to the FP, eFI has a fair sensitivity at 0.84 and a specificity of 0.78; these values were extracted directly from the study. The positive predictive value, however, is only just over half (0.52), indicative of a high number of false positives in practice. False negatives are likely minimised in this test as the negative predictive value (NPV) is 0.94; this test is likely good at ruling out frailty when it is not present, but it is only moderately good at identifying those with the condition.

### Claims-based frailty index

There were two studies [18, 19] which looked at a frailty index based on insurance claims data, the cFI. These were both USA-based and restricted their populations to individuals aged ≥65 and who were community-dwelling, and both used large studies to conduct their analysis (Health and Retirement Study and the Cardiovascular Health Study). Both had a similar mean age of participants and a similar proportion of women. Both also directly reported on the sensitivity and specificity with 95% confidence intervals calculated by Review Manager 5.4.1. Festa [18] determined an optimal threshold of 0.17 [18]; however, for the purposes of our study, we extracted data at the same threshold as Segal et al., which was 0.2 [19].

**Figure 2:**
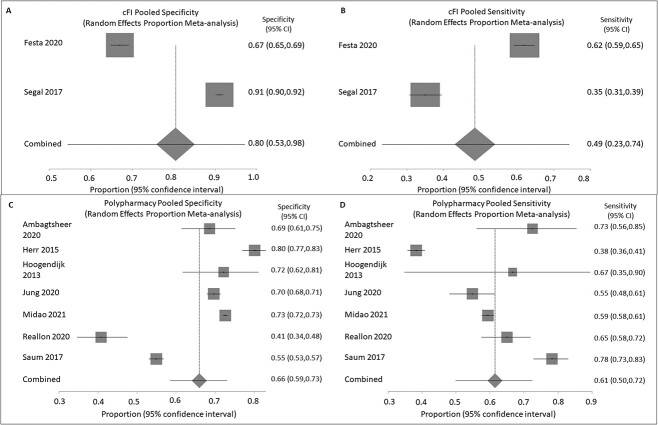
Random effects proportion meta-analysis of the reported sensitivities and specificities of studies using the cFI or polypharmacy as the index test and FP as the reference standard from StatsDirect. (A) Shows the specificity values for each cFI study with the 95% confidence intervals as determined by Review Manager 5.4.1 alongside the weights given to each study as demonstrated by the grey boxes. The pooled cFI specificity is estimated at 0.8 (95% CI = 0.53, 0.98), shown by the diamond. (B) Shows the same information as described in (A) for the sensitivity values given for the cFI studies with the pooled figure given as 0.49 (95% CI = 0.23, 0.74). (C) Shows the specificity values for each polypharmacy study with the 95% confidence intervals as determined by Review Manager 5.4.1 alongside the weights given to each study as demonstrated by the grey boxes. The pooled specificity for polypharmacy is estimated at 0.66 (95% CI = 0.59,0.73), shown by the diamond. (D) Shows the same information as described in (C) for the sensitivity values given for the same polypharmacy studies with the pooled figure given as 0.61 (95% CI = 0.50, 0.72).

In comparison to the FP, cFI had a pooled sensitivity of 0.48 and a specificity of 0.80 (Figure 2A and B), giving the test poor sensitivity and fair specificity overall.

### Polypharmacy

There were seven studies [20–26] that reported on polypharmacy. These studies gave the number of participants with polypharmacy who were frail compared to those with polypharmacy and not frail, allowing for the calculation of a sensitivity and specificity (Figure 2C and D) analysis. One study [21] did, however, have an issue with the missing data (see quality assessment), and this was overcome by extracting the most complete data. The studies were deemed similar enough to perform a pooled proportion analysis. Age for inclusion was varied, but the average ages were similar and all populations were community-based. They all also used the threshold of more than or equal to five medications to define polypharmacy, allowing for a pooled analysis.

**Figure 3:**
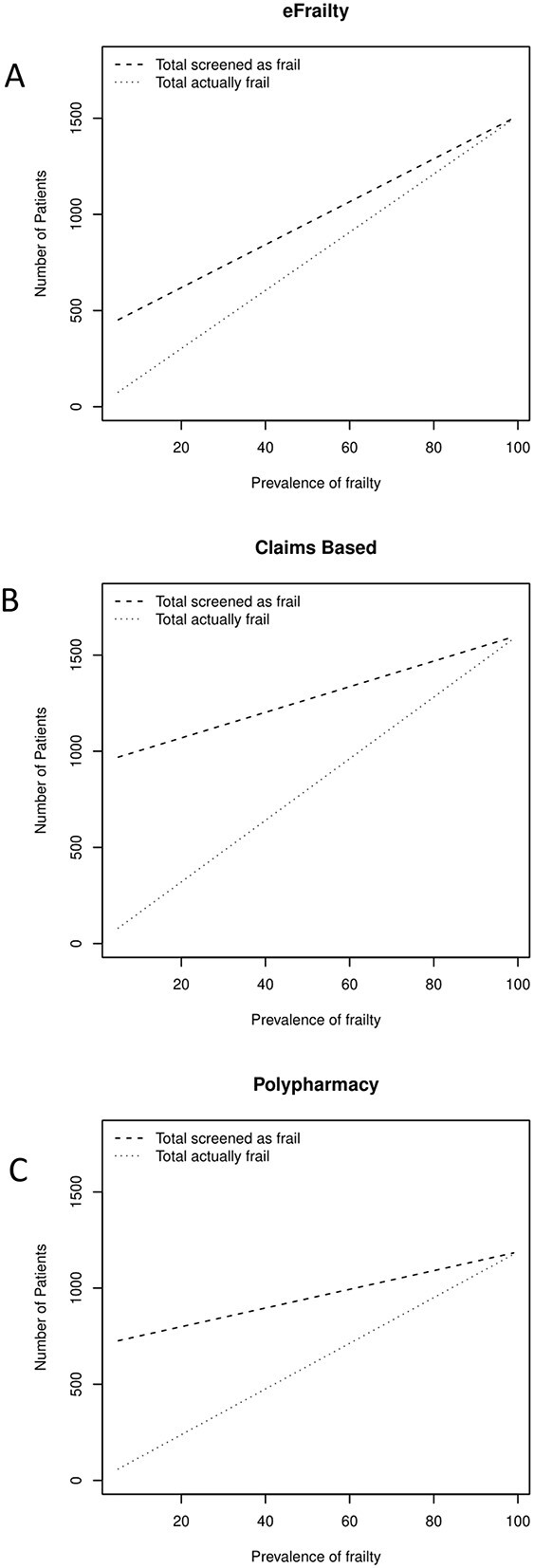
Correlation between the number of patients (aged ≥65) and the prevalence of frailty (%) on the performance of (A) eFI, (B) cFI and (C) polypharmacy.

The sensitivity and specificity values obtained were then used to model their application in a hypothetical UK GP practice with a list size of 10,000 of which 18% were over 65 years old [27] and 20% of those were frail [28]. Figure 3 shows the gap between those who are flagged as frail by the three index tests and those who are frail by the FP. The best-performing tool, the eFI [17] (Figure 3A), had a sensitivity of 85% (95% CI = 55–98%) and a specificity of 78% (95% CI = 64–89). For an average UK General Practice of 10,000, eFI would flag 597 as frail of whom 272 would be frail by the FP and an additional 51 frail patients would be missed. Both positive predictive value and NPV will predictably increase as the prevalence of frailty increases, demonstrated by the convergence of the index test and FP performance.

### Quality assessment (QUADAS-2)

Almost all studies had an unclear or high risk of bias. All polypharmacy papers were ruled as unclear for the applicability of index test due to these studies not proposing polypharmacy as a diagnostic tool. The full assessment is available in Supplementary Material 4.

Flow and timing domains were marked as high for Hoogendijk [22] and Jung [23] as it was not clear the arrangements for participants who could not perform physical assessments. It would be intuitive to assume that if an individual could not perform a test that they would score at the frailest end; but this might not be the case for all. For example, non-frail individuals who are wheelchair-bound cannot perform timed up and go but would score as “frail” on this metric. More transparency from these papers within the methodology is needed detailing how such participants would be managed. Ambagtsheer [20] also had a high risk of bias as they recruited only from a pool of patients who attended appointments at the GP practice. Herr [21] had a number of participants who were included within the initial frailty demographics analysis but were not within the multivariate analysis performed (*n* = 2,286 compared to *n* = 2,115), why this was the case was not presented in a transparent way by the study.

Many studies have resulted in the frailest participants being excluded. Most studies required the FP data to be complete, which may not have allowed those who could not perform the physical tasks to be included. This was overcome in some studies by self-report or multiple imputation. Festa and colleagues [18] used multiple imputation to overcome the missing values in their FP assessment and found that those participants were frailer than those with the complete data. From this, we produced a summary table to assess participant selection in further detail, which is available in Supplementary Material 4. It was also the case in half of the studies in which assessments did not take place within the home, thus essentially excluding house-bound individuals. There was also mixed reporting on the methods used to include participants who may lack capacity.

## Discussion

We identified three VEFMs that can be used to screen electronic primary care records and identify those with frailty from their electronic primary care records. One study [17] of 60 patients, average age 80.2 years, examined eFI, reporting a sensitivity of 0.84 (95% CI = 0.55, 0.98) and specificity of 0.78 (0.64, 0.89). Two studies [18, 19] of 7,679 patients, average age 75.5, examined cFI, with a pooled sensitivity of 0.84 (95% CI = 0.55, 0.98), and specificity of 0.78 (0.64, 0.89). Seven [20–26] studies of 34,328 patients, average age 77.4, examined a polypharmacy as a screening tool (defined as more than or equal to five medications) with a pooled sensitivity of 0.84 (95% CI = 0.55, 0.98), and specificity of 0.78 (0.64, 0.89). However, even the best-performing tool, the eFI, would have a false positive rate of nearly 50%, and this makes implementing a screening programme resource-intensive due to many individuals needing follow-up and additional testing.

There were more studies on eFI found in the search, but these were excluded as they assessed accuracy using statistical methods that are not useful for a diagnostic test accuracy review, for example, convergent validity [29] and hazard ratios [30].

These studies [29, 30], and others, treated frailty as a continuous variable, which may be more accurate, but this is of limited use to the clinician who needs to identify patients who are frail and to consider a cut-off. These methods either do not define a threshold or compare with an in-person assessment; many excluded studies did not define a cut-off or threshold for frailty. Given that we were only able to include this one small study on eFI due to this lack of cut-off, there is a need for further research and reporting of this in the future. This is particularly important within the UK setting, given its widespread use.

To our knowledge, this is the first review that focuses on the comparison between frailty tools which use primary care data and in-person assessment. Clegg and colleagues [9] also reviewed tools by their diagnostic test accuracy, but they focused on simple face-to-face measures. Ambagtsheer et al. [10] was another review of this type, and this included polypharmacy and we were able to include additional studies which have been published since. Ambagtsheer et al. [10] reported higher sensitivity and specificity than the pooled figures reported here and also compared to FP. It was suggested that an older population should be targeted to improve accuracy and that a two-step system could be implemented to improve diagnostic accuracy [9]. This fits in with our hypothetical GP practice model, and we would suggest that the use of older populations who typically have higher prevalence of frailty would increase the accuracy of identification. This review also adds to the work by Son Nghiem et al. [15], which focused on automated frailty measurement tools. Their comprehensive narrative synthesis highlighted the strong predictive ability of such tools of negative health outcomes, but moderate association with the FP. The application of different synthesis methods allows for our review to complement this existing work.

Though the sensitivity and specificity are considered in this work, it is important to consider the other principles of screening according to the UK Screening Committee [31]. This study starts to assess the interpretation of screening test results in our example practice. However, it does not address the acceptability of the test and the screening programme to professionals and the public, the availability of a treatment and whether there is a latent phase of the condition and the evidence of benefit for early identification [31].

Strengths of this review included a comprehensive search strategy and we used multiple methods of searching to ensure all relevant literatures were captured. We were able to pool the polypharmacy and the cFI studies to give an overall accuracy of sensitivity and specificity as these studies were sufficiently similar, and this makes the reported values likely more accurate than those previously reported.

The limitations of this review were that we were not able to include all studies found particularly on the eFI due to lack of reporting of binary cut-off points. Given more time and resources, it would also be possible to look at the individual patient data for these studies and define an optimal cut-off to maximise the sensitivity and specificity. A further limitation of some of the studies was that there were limited details on the accommodations made for those with limited mobility who may be missing the FP data, be house-bound or those with limited capacity. As one study discussed, this may be because they are simply too frail to perform the given tasks [18]. It is recommended that this should be considered in the frailty studies in the future. This also shows where data-based frailty tools are an advantage over physical measures as they give a holistic view of a person and require little to no effort from the participant.

## Conclusions and implications

Frailty screening using electronic primary care records is feasible and can allow for the early identification of frail individuals; however, the best available tool, eFI, could lead to a false positive rate of 47.6%. This requires improvement prior to the implementation of a universal screening programme.

## Supplementary Material

Supplementary-material_1_afad173

Supplementary_material_2_afad173

Supplementary_Material_3_afad173

Supplementary_material_4_afad173

## References

[1] Cesari M, Theou O. Frailty: The Broad View. In: Fillit H, Rockwood K, Young J (eds.) Brocklehurst’s Textbook of Geriatric Medicine and Gerontology, 8th edition. Philadephia: Elsevier Inc, 2017.

[2] Fried LP, Tangen CM, Walston J et al. Frailty in older adults: evidence for a phenotype. J Gerontol A Biol Sci Med Sci 2001; 56: M146–56. 11253156 10.1093/gerona/56.3.m146

[3] Rockwood K . A global clinical measure of fitness and frailty in elderly people. Can Med Assoc J 2005; 173: 489–95. 16129869 10.1503/cmaj.050051 PMC1188185

[4] Song X, Mitnitski A, Rockwood K. Prevalence and 10-year outcomes of frailty in older adults in relation to deficit accumulation. J Am Geriatr Soc 2010; 58: 681–7. 20345864 10.1111/j.1532-5415.2010.02764.x

[5] Keeble E, Roberts HC, Williams CD et al. Outcomes of hospital admissions among frail older people: a 2-year cohort study. Br J Gen Pract 2019; 69: e555–60. 31308000 10.3399/bjgp19X704621 PMC6650131

[6] NHS England . NHS England Standard General Medical Services Contract 2017/18. NHS England, 2018. [cited 3 November 2022]; https://www.england.nhs.uk/publication/nhs-england-standard-general-medical-services-contract-2017-18/ (3 November 2022, date last accessed).

[7] Walston J, Buta B, Qian-Li X. Frailty screening and interventions: considerations for clinical practice. Clin Geriatr Med 2018; 34: 25–38. 29129215 10.1016/j.cger.2017.09.004 PMC5726589

[8] McInnes MDF, Moher D, Thombs BD et al. Preferred reporting items for a systematic review and meta-analysis of diagnostic test accuracy studies: the PRISMA-DTA statement. JAMA 2018; 319: 388–96. 29362800 10.1001/jama.2017.19163

[9] Clegg A, Rogers L, Young J. Diagnostic test accuracy of simple instruments for identifying frailty in community-dwelling older people: a systematic review. Age Ageing 2015; 44: 148–52. 25355618 10.1093/ageing/afu157

[10] Ambagtsheer RC, Thompson MQ, Archibald MM, Casey MG, Schultz TJ. Diagnostic test accuracy of self-reported screening instruments in identifying frailty in community-dwelling older people: a systematic review. Geriatr Gerontol Int 2020; 20: 14–24. 10.1111/ggi.13810 31729157

[11] Drubbel I, Numans ME, Kranenburg G, Bleijenberg N, de Wit NJ, Schuurmans MJ. Screening for frailty in primary care: a systematic review of the psychometric properties of the frailty index in community-dwelling older people. BMC Geriatr 2014; 14: 27. 24597624 10.1186/1471-2318-14-27 PMC3946826

[12] Shashikumar SA, Huang K, Konetzka RT, Joynt Maddox KE. Claims-based frailty indices: a systematic review. Med Care 2020; 58: 815–25. 32520767 10.1097/MLR.0000000000001359

[13] Faller JW, Pereira D do N, de Souza S, Nampo FK, Orlandi F de S, Matumoto S. Instruments for the detection of frailty syndrome in older adults: a systematic review. PloS One 2019; 14: e0216166. 31034516 10.1371/journal.pone.0216166 PMC6488093

[14] Rodríguez-Laso Á, O’Caoimh R, Galluzzo L et al. Population screening, monitoring and surveillance for frailty: three systematic reviews and a grey literature review. Ann 1st Super Sanita 2018; 54: 253–62. 10.4415/ANN_18_03_13 30284553

[15] Nghiem S, Sajeewani D, Henderson K et al. Development of frailty measurement tools using administrative health data: a systematic review. Arch Gerontol Geriatr 2020; 89: 104102. 10.1016/j.archger.2020.104102. 32464423

[16] Whiting PF . QUADAS-2: a revised tool for the quality assessment of diagnostic accuracy studies. Ann Intern Med 2011; 155: 529. 22007046 10.7326/0003-4819-155-8-201110180-00009

[17] Ambagtsheer RC, Beilby J, Dabravolskaj J et al. Application of an electronic frailty index in Australian primary care: data quality and feasibility assessment. Aging Clin Exp Res 2019; 31: 653–60. 30132204 10.1007/s40520-018-1023-9

[18] Festa N, Shi SM, Kim DH. Accuracy of diagnosis and health service codes in identifying frailty in Medicare data. BMC Geriatr 2020; 20: 329. 32894057 10.1186/s12877-020-01739-w PMC7487915

[19] Segal JB, Huang J, Roth DL, Varadhan R. External validation of the claims-based frailty index in the national health and aging trends study cohort. Am J Epidemiol 2017; 186: 745–7. 28938711 10.1093/aje/kwx257 PMC5860423

[20] Ambagtsheer RC, Visvanathan R, Dent E et al. Commonly used screening instruments to identify frailty among community-dwelling older people in a general practice (primary care) setting: a study of diagnostic test accuracy. J Gerontol A Biol Sci Med Sci 2020; 75: 1134–42. 31689342 10.1093/gerona/glz260

[21] Herr M, Robine J-M, Pinot J et al. Polypharmacy and frailty: prevalence, relationship, and impact on mortality in a French sample of 2350 old people. Pharmacoepidemiol Drug Saf 2015; 24: 637–46. 25858336 10.1002/pds.3772

[22] Hoogendijk EO, van der Horst HE, Deeg DJH et al. The identification of frail older adults in primary care: comparing the accuracy of five simple instruments. Age Ageing 2013; 42: 262–5. 23108163 10.1093/ageing/afs163

[23] Jung H, Kim M, Lee Y, Won CW. Prevalence of physical frailty and its multidimensional risk factors in Korean community-dwelling older adults: findings from Korean Frailty and Aging Cohort Study. Int J Environ Res Public Health 2020; 17: 7883. 10.3390/ijerph17217883. PMC7662797 33121159

[24] Midão L, Brochado P, Almada M, Duarte M, Paúl C, Costa E. Frailty status and polypharmacy predict all-cause mortality in community dwelling older adults in Europe. Int J Environ Res Public Health 2021; 18: 3580. 10.3390/ijerph18073580. 33808273 PMC8036295

[25] Reallon E, Chavent B, Gervais F et al. Medication exposure and frailty in older community-dwelling patients: a cross-sectional study. Int J Clin Pharmacol 2020; 42: 508–14. 10.1007/s11096-020-01007-2 32140916

[26] Saum K-U, Schöttker B, Meid AD et al. Is polypharmacy associated with frailty in older people? Results from the ESTHER cohort study. J Am Geriatr Soc 2017; 65: e27–32. 28024089 10.1111/jgs.14718

[27] Public Health Scotland . Practice Populations (List Sizes) by Sex and Age Group. Information Services Division, National Services Scotland. https://www.isdscotland.org/Health-Topics/General-Practice/Workforce-and-Practice-Populations/_docs/Practice_ListSizes_Oct2021.xlsx?14:19:07 (16 December 2021, date last accessed).

[28] O’Caoimh R, Sezgin D, O’Donovan MR et al. Prevalence of frailty in 62 countries across the world: a systematic review and meta-analysis of population-level studies. Age Ageing 2021; 50: 96–104. 33068107 10.1093/ageing/afaa219

[29] Brundle C, Heaven A, Brown L et al. Convergent validity of the electronic frailty index. Age Ageing 2019; 48: 152–6. 30321256 10.1093/ageing/afy162

[30] Clegg A, Bates C, Young J et al. Development and validation of an electronic frailty index using routine primary care electronic health record data. Age Ageing 2016; 45: 353–60. 26944937 10.1093/ageing/afw039 PMC4846793

[31] Criteria for Appraising the Viability, Effectiveness and Appropriateness of a Screening Programme. Gov.uk, London: National Screening Committee. https://www.gov.uk/government/publications/evidence-review-criteria-national-screening-programmes/criteria-for-appraising-the-viability-effectiveness-and-appropriateness-of-a-screening-programme (16 December 2021, date last accessed).

