# Supplementary material for: Validated frailty measures using electronic primary care records: a review of diagnostic test accuracy
Source: Age Ageing. 2023 Nov 17;52(11):afad173. doi: 10.1093/ageing/afad173 (PMC10873280; doi:10.1093/ageing/afad173)
Supplement: Supplementary-material_1_afad173 [file supplementary-material_1_afad173.docx]

**Supplementary Material 1: Search Terms**

***Applied to Ovid (MEDLINE, Embase and Cochrane Central Register of Controlled Trials) and CIHNAL:***

1. Frailty/ or Frail Elderly/

2. Geriatric Assessment/ or frailty assessment.mp.

3. exp Medical Records/

4. Primary Health Care/

5. exp Data Collection/

6. 3 or 4 or 5

7. "Sensitivity and Specificity"/

8. sensitivit*.tw.

9. specificit*.tw.

10. predictive value.tw.

11. Diagnosis, Differential/

12. accuracy.mp.

13. "Reproducibility of Results"/

14. Mortality/

15. Survival Rate/

16. Hospitalization/

17. Long-Term Care/

18. Residential Facilities/

19. 7 or 8 or 9 or 10 or 11 or 12 or 13 or 14 or 15 or 16 or 17 or 18

20. 1 and 2 and 6 and 19

***Applied to Pubmed:***

(((((sensitivity and specificity OR sensitivit* OR Specificit* OR predictive value OR diagnosis OR accuracy OR reproducibility of results OR mortality OR survival rate OR hospitalization OR long term care OR residential facilities) AND (Geriatric assessment OR frailty assessment)) AND (Medical records OR primary health care OR Data collection)) AND (Frail OR frail elderly)) NOT ("systematic review"[All Fields])) NOT ("review"[All Fields])
