# Supplementary material for: Validated frailty measures using electronic primary care records: a review of diagnostic test accuracy
Source: Age Ageing. 2023 Nov 17;52(11):afad173. doi: 10.1093/ageing/afad173 (PMC10873280; doi:10.1093/ageing/afad173)
Supplement: Supplementary_material_2_afad173 [file supplementary_material_2_afad173.docx]

**Supplementary Material 2: Data Collection Form**

| **Administrative Details** | | | | |
| --- | --- | --- | --- | --- |
| Study ID |  | | | |
| Publication type |  | | | |
| Funding |  | | | |
| Country |  | | | |
| Language |  | | | |
| Author contact info |  | | | |
| **Study Details** | | | | |
| Aim |  | | | |
| Design |  | | | |
| Setting | |  | | |
| Duration |  | | | |
| Statistical analysis |  | | | |
| Sample identification |  | | | |
| Eligibility criteria | Inclusion | |  | |
|  | Exclusion | |  | |
| **Comparator** | | | | |
| Screening tool |  | | | |
| Which data was used |  | | | |
| Control/comparator |  | | | |
| Follow-up period |  | | | |
| **Participants** | | | | |
| Number |  | | | |
| Missing Data |  | | | |
| Age (years) (mean/median, SD/range) |  | | | |
| Gender (M/F), n (%) |  | | | |
| Comorbidities, n |  | | | |
| **Outcomes** | | | | |
| Number Frail | | Index Test | |  |
|  |  | Reference Standard | |  |
| Primary | | | | |
| Sensitivity | |  | | |
| Specificity | |  | | |
| PPV | |  | | |
| NPV | |  | | |
| TP | |  | | |
| TN | |  | | |
| Secondary | | | | |
|  | Index Test | | | Reference Standard |
| Mortality |  | | |  |
| Hospitalisation |  | | |  |
| Admission to institution |  | | |  |
| Other adverse health outcomes |  | | |  |
| Any other effectiveness measures |  | | |  |
| Quality Measurement | | | | |
| Patient Selection (methods of, sample type, case-control avoided?, who was excluded and included?) | |  | | |
| If Primary outcomes reported | | | | |
| Reference standard (likely to correctly classify the condition, interpreted without knowledge of results of index test, does the condition identified match the review question) | |  | | |
| Secondary outcomes | | | | |
| Test (pre-specified threshold, interpretation different from the review question, likely to correctly classify, interpreted independently, condition identified matches the review question) | |  | | |
| Flow and timing (description of those excluded, time interval between tests, did all patients receive the same reference standard, were all included in analysis) | |  | | |
