# Supplementary material for: Validated frailty measures using electronic primary care records: a review of diagnostic test accuracy
Source: Age Ageing. 2023 Nov 17;52(11):afad173. doi: 10.1093/ageing/afad173 (PMC10873280; doi:10.1093/ageing/afad173)
Supplement: Supplementary_Material_3_afad173 [file supplementary_material_3_afad173.docx]

**Supplementary Material 3: Two-By-Two Tables by Study**

| **Study ID** | **TP** | **FP** | **FN** | **TN** |
| --- | --- | --- | --- | --- |
| **Ambagtsheer 2019 [15]** | 11 | 10 | 2 | 37 |
| **Ambagtsheer 2020 [16]** | 29 | 59 | 11 | 129 |
| **Festa 2020 [17]** | 742 | 627 | 454 | 1274 |
| **Herr 2015 [18]** | 566 | 144 | 912 | 582 |
| **Hoogendijk 2013 [19]** | 8 | 25 | 4 | 65 |
| **Jung 2020 [20]** | 125 | 812 | 103 | 1867 |
| **Midao 2021 [21]** | 1804 | 5925 | 1233 | 15731 |
| **Reallon 2020 [22]** | 117 | 132 | 63 | 91 |
| **Saum 2017 [23]** | 212 | 1255 | 59 | 1532 |
| **Segal 2017 [24]** | 171 | 357 | 318 | 3608 |
