# Supplementary material for: Validated frailty measures using electronic primary care records: a review of diagnostic test accuracy
Source: Age Ageing. 2023 Nov 17;52(11):afad173. doi: 10.1093/ageing/afad173 (PMC10873280; doi:10.1093/ageing/afad173)

**Appendix 4: QUADAS-2 assessment and assessment of patient inclusion methods**

| ***Study*** | ***Completion reported? (%)*** | ***Participants unable to mobilise included?*** | ***Participants unable to do grip strength included?*** | ***Patients without capacity to consent included?*** | ***Housebound patients included?*** |
| --- | --- | --- | --- | --- | --- |
| **Ambagtsheer 2019 [15]** | **✓** | **X** | **X** | **X** | **X** |
| **Ambagtsheer 2020 [16]** | **✓** | **X** | **X** | **✓** | **X** |
| **Festa 2020 [17]** | **✓** | **✓** | **✓** | **✓** | **✓** |
| **Herr 2015 [18]** | **✓** | **✓** | **✓** | **?** | **✓** |
| **Hoogendijk 2013 [19]** | **✓** | **X** | **X** | **?** | **?** |
| **Jung 2020 [20]** | **✓** | **X** | **X** | **X** | **X** |
| **Midao 2021 [21]** | **✓** | **✓** | **✓** | **✓** | **✓** |
| **Reallon 2020 [22]** | **✓** | **X** | **X** | **?** | **X** |
| **Saum 2017 [23]** | **✓** | **✓** | **✓** | **X** | **✓** |
| **Segal 2017 [24]** | **✓** | **X** | **X** | **✓** | **✓** |

| **✓ Confirmed** | **X Not Confirmed** | **? Unclear Reporting** |
| --- | --- | --- |


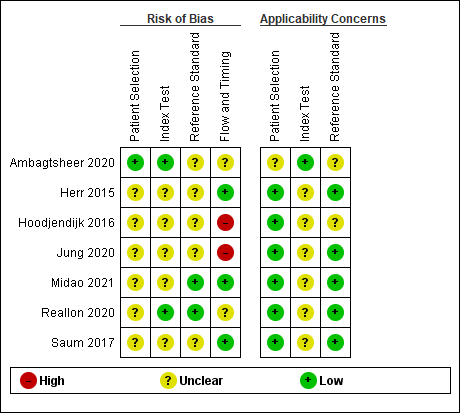

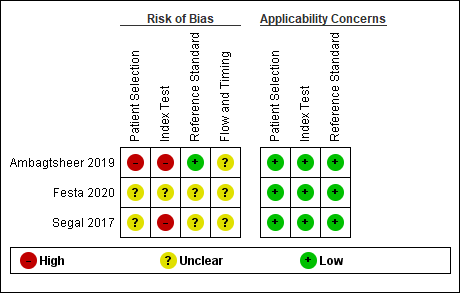

Supplement: Supplementary_material_4_afad173 [file supplementary_material_4_afad173.docx]
